# Cholesterol Homeostasis: An In Silico Investigation into How Aging Disrupts Its Key Hepatic Regulatory Mechanisms

**DOI:** 10.3390/biology9100314

**Published:** 2020-09-30

**Authors:** Amy Elizabeth Morgan, Mark Tomás Mc Auley

**Affiliations:** Faculty of Science and Engineering, University of Chester, Thornton Science Park, Chester CH2 4NU, UK; amy.morgan@chester.ac.uk

**Keywords:** aging, cholesterol biosynthesis, reactive oxygen species, mathematical model, systems biology

## Abstract

**Simple Summary:**

Cardiovascular disease is the leading cause of death worldwide. High blood cholesterol levels are associated with an increased risk of this condition. How high levels of blood cholesterol result in cardiovascular disease during aging is a difficult question to answer. Computational models have been used over the years to help understand this problem, as they are capable of representing complex systems such as this. The aim of this work was to use a computational model of cholesterol metabolism to better understand why, in certain studies involving the oldest old (persons ≥ 85 years), high blood cholesterol levels have been associated with a decreased risk of death. Using our computational model, we found that key age-associated changes to how cholesterol is processed in the liver could be responsible for this observation. The findings from our work contribute to our understanding of cholesterol metabolism in older people and how treatments could be developed in the future to promote healthy aging.

**Abstract:**

The dysregulation of intracellular cholesterol homeostasis is associated with several age-related diseases, most notably cardiovascular disease (CVD). Research in this area has benefitted from using computational modelling to study the inherent complexity associated with the regulation of this system. In addition to facilitating hypothesis exploration, the utility of modelling lies in its ability to represent an array of rate limiting enzymatic reactions, together with multiple feedback loops, which collectively define the dynamics of cholesterol homeostasis. However, to date no model has specifically investigated the effects aging has on this system. This work addresses this shortcoming by explicitly focusing on the impact of aging on hepatic intracellular cholesterol homeostasis. The model was used to investigate the experimental findings that reactive oxygen species induce the total activation of 3-hydroxy-3-methylglutaryl-coenzyme A (HMG-CoA) reductase (HMGCR). Moreover, the model explored the impact of an age-related decrease in hepatic acetyl-CoA acetyltransferase 2 (ACAT2). The model suggested that an increase in the activity of HMGCR does not have as significant an impact on cholesterol homeostasis as a decrease in hepatic ACAT2 activity. According to the model, a decrease in the activity of hepatic ACAT2 raises free cholesterol (FC) and decreases low-density lipoprotein cholesterol (LDL-C) levels. Increased acetyl CoA synthesis resulted in a reduction in the number of hepatic low-density lipoprotein receptors, and increased LDL-C, FC, and cholesterol esters. The rise in LDL-C was restricted by elevated hepatic FC accumulation. Taken together these findings have important implications for healthspan. This is because emerging clinical data suggest hepatic FC accumulation is relevant to the pathogenesis of non-alcoholic fatty liver disease (NAFLD), which is associated with an increased risk of CVD. These pathophysiological changes could, in part, help to explain the phenomenon of increased mortality associated with low levels of LDL-C which have been observed in certain studies involving the oldest old (≥85 years).

## 1. Introduction

Cholesterol is an important macronutrient which has several important roles within the body. It consolidates cell membranes, is the precursor of steroid hormone synthesis, is a prerequisite for vitamin D synthesis, and is essential to bile acid production [1,2,3,4,5]. However, the dysregulation of cholesterol metabolism, which leads to hypercholesterolemia, is inexorably associated with the pathophysiology of several diseases; the most notable being cardiovascular disease (CVD) [6,7,8,9,10]. Elevated plasma cholesterol, particularly low-density lipoprotein cholesterol (LDL-C) is firmly established as an independent risk factor for atherosclerotic CVD [11,12]. Aging has a pleiotropic effect on cholesterol metabolism, altering many of the key processes responsible for its regulation [13,14,15,16,17,18,19,20,21]. It is therefore unsurprising that population studies have shown that LDL-C increases in both males and females up until the midpoint of life [22,23,24,25]. Paradoxically, it has been found in population studies that LDL-C gradually decreases in the latter decades of life [26,27,28,29]. To date, a conclusive mechanistic explanation for this conundrum remains elusive. However, it is possible this is simply a survivor effect. Studies in the oldest old (persons ≥ 85 years) also reveal intriguing questions about cholesterol metabolism and aging. For instance, it has been observed that both high and low levels of LDL-C can have a similar impact on mortality risk in this population group [30]. This was revealed despite CVD being the main cause of mortality among this cohort. In light of findings such as this, others have challenged scientific orthodoxy by suggesting high levels of LDL-C could be cardioprotective against infections and atherosclerosis [31,32,33,34]. However, it is challenging to see how this is plausible mechanistically. An alternative explanation for these findings could reside within the biochemical mechanisms which govern hepatic cholesterol homeostasis, and how these processes are affected by aging [35,36].

The liver is the focal point of cholesterol homeostasis, synthesizing cholesterol and triglycerides to be incorporated into very low-density lipoproteins (VLDLs) [37]. VLDLs are hydrolysed in the plasma, by lipoprotein lipase into LDL, via intermediate-density lipoproteins [38]. Circulating LDL-C is removed by LDL receptors (LDLrs), in a process which is regulated by cellular sterol concentrations [39]. An increase in cellular cholesterol results in the activation of insulin-induced genes (Insigs) proteins, which bind to sterol regulatory element binding protein (SREBP) cleavage-activating protein (SCAP) in the endoplasmic reticulum (ER) [40], impeding the transfer of the SCAP/SREBP complex to the Golgi [41]. As the levels of sterol diminish, SREBP-2 is transferred to the Golgi where it is cleaved by subtilisin kexin isozyme/site-1 protease (SKI-1/S1P) [41,42], and the intramembranous metalloprotease site-2 protease (S2P). This extricates the membrane bound NH-terminal domain of SREBP-2. Two N-terminal fragments dimerise, then engage with importin-β, prior to their entry into the nucleus, whereby they induce SREBP-2-regulated gene promoters. This regulatory landscape also includes factors which control LDLr synthesis. Nuclear SREBP-2 raises the transcription levels of proprotein convertase subtilisin/kexin type 9 (PCSK9) [43]. PCSK9 reduces the number of LDLr by elevating their metabolism, and degradation, inhibiting LDL uptake [44]. Elevated intracellular cellular cholesterol levels subdue SREBP-2 release from the ER, thus PCSK9 transcription is reduced, which raises LDLr levels [45]. The coordinated synergy of SREBP-2-induced transcription of both LDLr and PCSK9 modulates LDL-C concentration [46]. Moreover, LDL-derived cholesterol provokes hepatic acyl CoA: cholesterol acyltransferase (ACAT2) [47]. This enzyme catalyses free cholesterol (FC) to cholesterol esters (CE).

In this work, a previously enunciated hypothesis is outlined and examined [35]. It is hoped the examination of this idea will help to explain why low levels of LDL-C have been associated with an increased rate of mortality in the oldest old. The premise of the hypothesis is as follows: it has been found experimentally that a decrease in hepatic cellular antioxidant capacity occurs during aging [48,49]. This deficiency opens up an avenue for hepatic reactive oxygen species (ROS) to induce the total activation of 3-hydroxy-3-methylglutaryl-coenzyme A (HMG-CoA) reductase (HMGCR), the key rate limiting enzyme in cholesterol biosynthesis [50,51,52,53,54]. Recent experimental findings add weight to this idea. Seo et al. (2019) found that treating rat HepG2 cells with 500 μM H_2_O_2_ led to a significant increase in SREBP2 and HMGCR [55]. An increase in cholesterol synthesis in turn elicited a corresponding rise in hepatic FC. This finding resonates with other work of this nature. For instance, it is important to note that hepatic FC has been observed to significantly rise with age in rats [56]. In some studies this has been observed to occur side by side with a reduction in HMGCR levels/activity [57]. Moreover, in certain studies it has been found that there is no difference between hepatic FC levels in young and old rats [17]. Taking account of these caveats it is possible to meaningfully speculate that due to a ROS-induced increase hepatic FC and an age-associated decrease in the activity of cholesterol-esterifying enzyme, ACAT2, there is restricted conversion of excess FC to CE [58]. However, it must be stressed that there is limited experimental evidence to support a reduction in ACAT2. The work that does support the notion was conducted using rabbits deficient in LDLrs. Despite this drawback, for the purposes of our hypothesis, and in line with the discovery-based exploratory nature of this work, we make the assumption that there is a decrease in hepatic ACAT2. If this does occur it can be reasonably inferred that the secretion of VLDL-C drops, and there is a corresponding decrease in LDL-C. As intracellular levels of FC accumulate, this state progresses to a pathophysiological condition akin to nonalcoholic fatty liver disease (NAFLD) [59]. Due to the strong association between NAFLD and increased risk of CVD [60], it is possible a deleterious condition akin to NAFLD, contributes to a pro-inflammatory state, which has the potential to explain why low levels of LDL-C have been associated with an increased rate of mortality in the oldest old.

Due to its ability to handle the complexities associated with aging and its adeptness at representing the processes associated with intracellular cholesterol homeostasis, computational systems biology offers an ideal framework for testing the hypothesis outlined in the previous paragraph [61,62,63,64,65,66,67,68,69,70,71,72,73,74]. Moreover, there is a growing momentum to better understand the aetiology of diseases such as CVD using computational modelling [75,76,77,78]. CVD is underscored by atherosclerosis, an inflammatory process whereby the LDL-C plays a key role. Due to the complex nature of CVD, there is a biomedical imperative to use models to help better understand what drives its onset and how cholesterol modulates this process. A plethora of models of cholesterol metabolism have been assembled previously [64,65,79,80,81,82,83,84,85,86,87,88,89,90,91]. These models have made invaluable contributions to the understanding of cholesterol metabolism. In particular, the models of Mazein et al. (2013), Watterson et al. (2013), and Benson et al. (2017), have laid the foundations for modelling the key regulatory processes which govern cholesterol biosynthesis using a systems biology framework and discipline specific standards [82,89,90]. Importantly, these models examined the cholesterol biosynthesis pathway and how its regulatory points can be used as targets for pharmaceutical interventions which help to maintain cholesterol homeostasis and reduce CVD risk. Other mathematical models have also adopted a systems biology and discipline-specific standards to conduct work of this nature. Specifically, they have focused on the intersection between cholesterol metabolism atherosclerosis. These include Gomez-Cabrero (2011), Parton et al. (2019), and Bekkar et al. (2018) [92,93,94]. However, to date no model has specifically investigated the effects aging has on hepatic intracellular cholesterol homeostasis. This work addresses this shortcoming by developing a model to explicitly focus on the impact of aging on hepatic intracellular cholesterol homeostasis. This ordinary differential equation (ODE) model includes the experimental finding that ROS induce the total activation of HMGCR. The model was then used to explore the effect of aging on hepatic cholesterol biosynthesis.

## 2. Materials and Methods

### 2.1. Network Diagram Construction and Model Assembly

**Figure 1:**
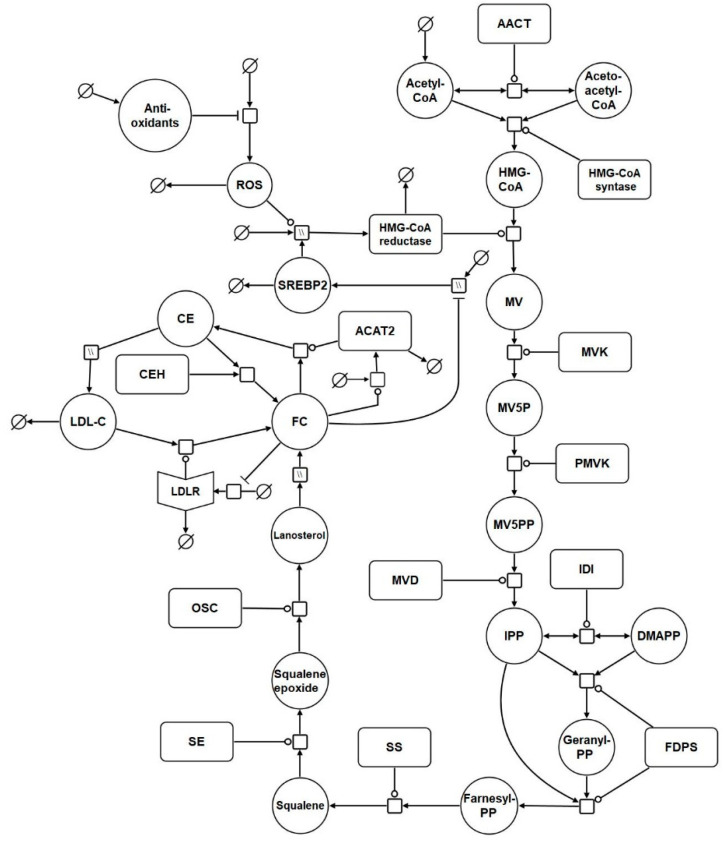
Network diagram of the model. The systems biology graphical notation network (SBGN) diagram comprises of key elements of the cholesterol biosynthesis pathway, and includes reactions which represent the synthesis, degradation, and regulation of hepatic LDL receptors (LDLrs). Finally, it incorporates reactions which depict the effects of aging on cholesterol homeostasis. Round arrow heads represent the target of a catalytic enzyme. Arrows represent flux. The mathematical symbol for an empty set represents a synthesis or sink reaction. All of these reactions are described in detail in the main body of the text. Hatched process nodes represent omitted processes.

Based on the reaction list in Table 1, a systems biology graphical notation network (SBGN) diagram was generated to visually capture the model reactions (Figure 1) [95]. SBGN is a framework which has been developed in order to capture biological networks in a clear and unambiguous manner and it is supported by a number of software packages [95]. In this case VANTED was used to create this the SBGN diagram [96] (Supplementary File S1). The add-on SBGN-ED can be used to export the map into SBGN markup language (SBGN-ML) [97].

A full list of the abbreviations used in Table 1 and Figure 1 is provided in Table S1 of Supplementary File S2. The model was assembled using the computational systems biology software tool COPASI [28]. COPASI was chosen due to its suitability for modelling systems of this nature, its intuitiveness, and the fact it is non-commercial software. In addition, COPASI supports the exchange schema systems biology markup language (SBML), which means the model is straightforward to update and adapt in the future (Supplementary Files S3 and S4).

The model commences with cholesterol biosynthesis. Firstly, acetyl CoA enters the biosynthesis pathway. Following this, acetoacetyl CoA thiolase catalyses the interconversion of acetyl CoA and acetoacetyl CoA. Acetyl CoA and acetoacetyl CoA undergo a condensation reaction to form 3-hydroxy-3-methylglutaryl-coenzyme A (HMG-CoA). Following this HMG-CoA is then converted by HMGCR to mevalonate. Phosphorylation of mevalonate forms mevalonate-5P, which is further phosphorylated to mevalonate-5PP. Decarboxylation and dehydration of mevalonate-5PP forms isopentenyl-PP (IPP), which converts to its isoform dimethylallyl-PP (DMAPP). DMAPP reacts with IPP to create geranyl-PP. Further condensation and the addition of another IPP creates farnesyl-PP. Condensation of 2 farnesyl-PP molecules forms squalene, which is converted to squalene epoxide before undergoing cyclisation to form lanosterol. Lanosterol is converted to FC, by a series of reactions. It is then converted to CE by ACAT2. This reaction is reversible and cholesteryl ester hydrolase (CEH) converts CE back to FC. The network diagram includes a nominal reaction which represents the putative effects of ROS on the model HMG-CoA. It also includes antioxidant levels which influence ROS levels.

### 2.2. Model Analysis

Values for kinetic parameters are contained in the Supplementary File S5. Many experimental values derived from the literature had to be adjusted to enable the model to enter steady state. The initial value references are also provided in Supplementary File S5. When this was done, COPASI identified a steady state which was asymptotically stable. Figure S1 in Supplementary File S2 illustrates model variables reaching the steady state. Once it was clear the model entered a steady state, a metabolic control analysis (MCA) was conducted [98]. MCA is a computational procedure which investigates the regulation and control of metabolic pathways [99]. It focuses on the elements which impact the flux of biochemical reaction networks. Once a model steady state is determined, an MCA can be conducted to quantify the extent to which metabolite steady-state concentrations depend on the velocity of certain reactions, or the concentration of particular variables. Thus, the MCA was completed in order to determine the effect changing different reactions had on steady state. There are two kinds of control coefficients: (1) the flux control coefficients and (2) the concentration control coefficients. A flux control coefficient is defined as the ratio between the relative change in the steady flux of a reaction and the relative change in the particular enzyme which is perturbed [100].

(1) $$C_{i}^{J}\underset{∆v_{i}\to 0}{\lim}\frac{∆J/J}{∆v_{i}/v_{i}}=\frac{dJ/J}{dv_{i}/v_{i}}=\frac{d\ln\left(J\right)}{d\ln\left(i\right)}$$

MCA can deal with local behaviour: the behaviour of a single step considered in isolation, and global behaviour: the behaviour of the step within the context of biochemical reaction network. Concentration control coefficients are a global property of the system and can be defined as the ratio between the relative change in the steady-state concentration of a given species and the relative change in the activity of the enzyme which is perturbed [100]:

(2) $$C_{i}^{S}\underset{∆v_{i}\to 0}{\lim}\frac{∆S/S}{∆v_{i}/v_{i}}=\frac{dS/S}{dv_{i}/v_{i}}=\frac{d\ln\left(S\right)}{d\ln\left(i\right)}$$

The greater the control coefficients, the greater the control species or parameter has over a particular variable. Thus, the goal was to use this technique as a means of determining the sensitivity of the model. It is important to note control coefficients may be negative or positive. If a value is negative it means that if the concentration of a particular species is increased, it will reduce the flux of a particular reaction.

The MCA was conducted in COPASI by selecting the following: “Tasks” - > “Metabolic Control Analysis”- > “Run”. An output file was generated which provided the results of the analysis. Supplementary File S2, which contains Tables S2 and S3, provides the full results of the MCA.

### 2.3. Hypothesis Exploration

The hypothesis outlined in the introduction was tested. To do this we examined if an increase in HMGCR activity influenced hepatic cholesterol and LDL-C levels. The baseline V_max_ for R4 was 1.43 µMoles/min, and it was necessary to increase this value to investigate the hypothesis. To do this, a parameter scan was conducted in COPASI, with integers between 1 and 10 analysed. The parameter scan in COPASI was conducted as follows: “Tasks”- >“Parameter Scan” - > “create”. Next “Reactions” was selected and then “Reaction Parameters”. The parameter to be scanned was selected, and “OK” was clicked resulting in a scan window appearing. In the “Intervals” box, a “min” and “max” for the scan was set and “Run” was selected to conduct the scan. Results were recorded at 3000 min (50 h), as a recent in vitro study indicated ROS increase cholesterol synthesis within this time period. Specifically, Seo et al. (2019) demonstrated that treating HepG2 cells with 500 μM H_2_O_2_ results in a significant increase in SREBP2, HMGCR and intracellular cholesterol levels [55].

The next aspect of the hypothesis which was examined, centred on the impact of a decline in ACAT2 with age. To investigate if a reduction in ACAT2 impacted cholesterol homeostasis, a parameter scan of the V_max_ for R15 was conducted. As the baseline V_max_ value was 0.04 µMoles/min, values between 0.01 and 0.06 µMoles/min were scanned using a step size of 0.01 and results were recorded at 3000 min.

It was important to determine the combined effect of these age-related changes. A parameter scan was conducted for the two parameters simultaneously. The V_max_ for R4 was scanned between 1 and 5 µMoles/min (integers). The V_max_ for R15 was scanned at 5 points between 0.01 and 0.05 µMoles/min. Results were recorded at 3000 min.

Based on the results of the MCA, it was found that reaction fluxes and species were most sensitive to perturbations in R1, acetyl CoA synthesis. This was considered an interesting finding because it is known that acetyl CoA can change significantly in response to a variety of pathological conditions [101]. Given that cholesterol is generated from acetyl CoA this had important implications for testing our hypothesis because increased acetyl CoA levels could be the key driver of an increase in hepatic FC rather than an increase in HMGCR activity. In fact, Seo et al. (2019) also found that acetyl CoA increased significantly when the primary hepatocyte cells HepG2 were treated with ROS. It was suggested that the results support the idea that ROS play an important role in perturbing cholesterol metabolism and consequently contribute to the accumulation of cholesterol hepatically during aging. Therefore, it was deemed cogent to explore in greater depth the effect of an increase in acetyl CoA synthesis has on the system.

To replicate an increase in acetyl CoA in a manner comparable to in vivo experimental work, the findings of Perry et al. (2017) were utilised [102]. Perry et al. (2017) outlined the effect of 3 conditions on male rat acetyl CoA levels: namely (1) insulin resistance, induced via a high fat diet, (2) high fat diet and T2DM, induced through the administration of 80 mg/kg nicotinamide followed by a low dose (40 mg/kg) of streptozotocin which resulted in whole-body insulin resistance and modest insulinopenia, and (3) T1DM, induced by the administration of 65 mg/kg streptozotocin. The authors reported a ~15%, ~75% and ~100% increase in acetyl CoA when compared to control animals. Therefore, the rate constant K_1_ for acetyl CoA synthesis was increased from 0.1 to 0.115, 0.175 and 0.2 respectively. Firstly, this simulation was conducted using the baseline age of the model. Secondly, the effect of increased acetyl CoA synthesis was investigated when aging was also simulated. To mimic the aging effect, the V_max_ for R4 was doubled to 2.86 µMoles/min, while the V_max_ for R15 was halved to 0.02 µMoles/min. This was to represent the ROS-induced increase in HMGCR activity and the decline in ACAT2 activity. Results were recorded at 3000 min.

## 3. Results

### 3.1. The ROS and HMG-CoA Reductase Hypothesis

**Figure 2:**
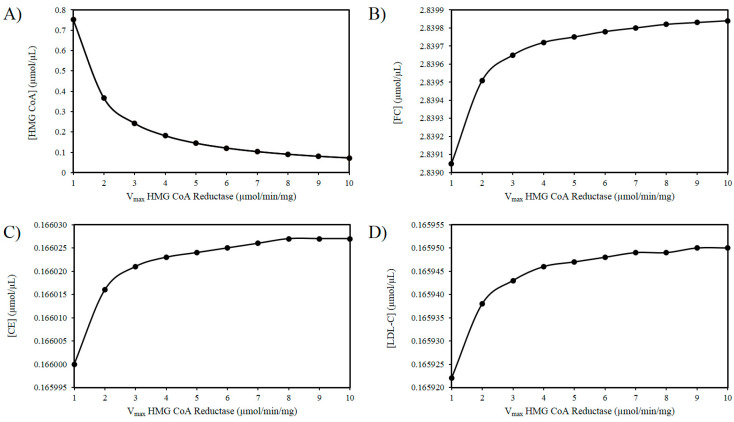
Effect of HMG-CoA reductase (HMGCR) modulation on (**A**) HMG-CoA, (**B**) FC, (**C**) LDL-C, and (**D**) CE.

As outlined above experimental findings suggest ROS increases the activity of HMGCR. The mechanism(s) responsible for this effect remain to fully delineated, however, if the activity of HMGCR increases, it is not improbable to assume that there will be an increase in the V_max_ of this enzyme [103]. Thus, using these in silico experiments, the effect of increasing ROS levels was modelled by elevating the V_max_ of HMGCR. Figure 2A illustrates that as the V_max_ of HMGCR increases there is a drop in HMG-CoA. Figure 2B shows a progressive accumulation of FC with increasing V_max_. Figure 2C shows a very slight increase in CE as a result of this analysis. Similarly, there is a negligible increase in LDL-C as the V_max_ of HMGCR increases (Figure 2D).

### 3.2. Exploring the Effects of a Decrease in ACAT2 Activity with Age

**Figure 3:**
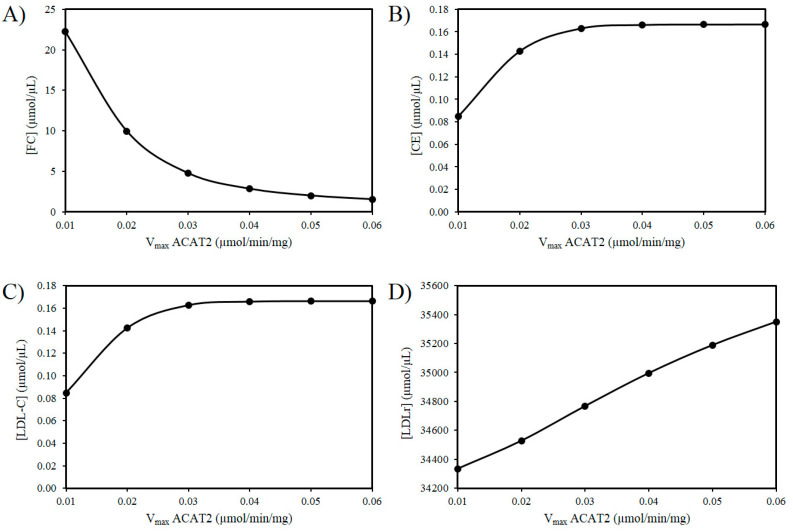
Effect of ACAT2 modulation on (**A**) FC, (**B**) CE, (**C**) LDL-C, and (**D**) LDLr.

Next the effects of reducing the activity of ACAT2 were investigated. An age-related decrease in ACAT2 is very tentatively supported by the work of Shiomi et al. (2000) who found that homozygous Watanabe heritable hyperlipidemic rabbits; deficient in LDLrs had decreased ACAT2 activity with age [58]. According to our hypothesis, it is possible that a decrease in hepatic ACAT2 activity can result in the accumulation of hepatic FC. Figure 3A reveals an increase in FC as the V_max_ of ACAT2 decreases. Additionally, based on this idea there should be a corresponding decrease in CE and LDL-C as the activity of ACAT2 decreases (Figure 3B,C). Finally, as the V_max_ for ACAT2 declines there is corresponding decrease in LDLr numbers (Figure 3D).

### 3.3. ROS Combined with a Decrease in ACAT2 with Age

The next in silico experiment centred on investigating the effect of ROS in conjunction with changes to the activity of ACAT2 (Table 2). Figure S2 in Supplementary File S2 shows how these combined aging effects impact LDL-C levels in the model. Modulation of the V_max_ for ACAT2 had a more pronounced effect on FC, CE, LDL-C, and LDLr than perturbation of V_max_ for HMGCR. Specifically, there is a negligible increase in FC and CE as V_max_ for HMGCR is increased, while a reduction in ACAT2 has a more prominent effect in reducing CE, and elevating FC (Figure S2A,B). Additionally, increased V_max_ for HMGCR produced a minimal rise in LDL-C whilst negligibly decreasing LDLr numbers. This was associated with the concomitant decline in LDL-C and LDLr numbers as a result of a reduced V_max_ for ACAT2 (Figure S2C,D).

### 3.4. The Impact of Increasing Acetyl CoA Synthesis

**Figure 4:**
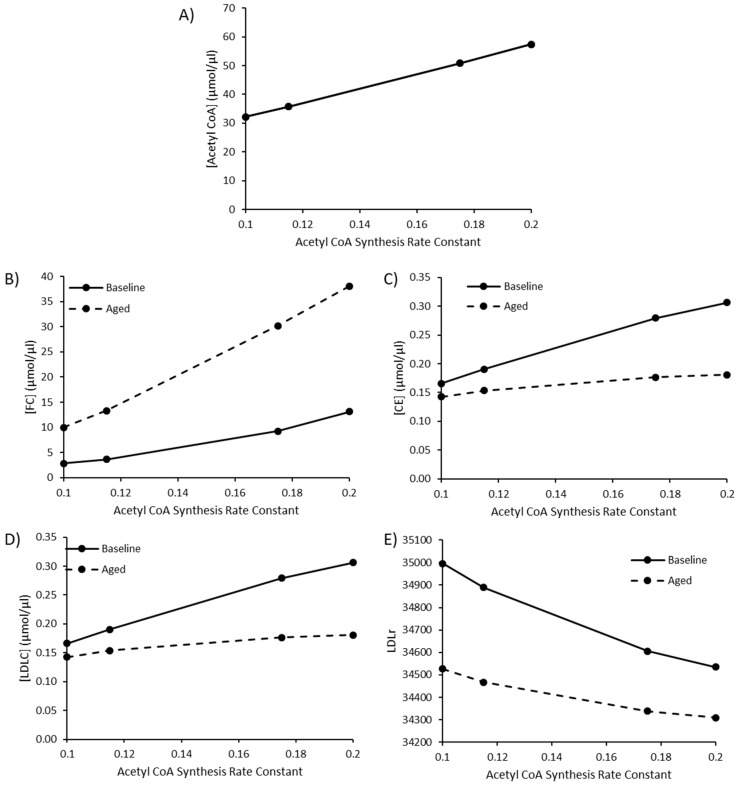
The combined effect of elevated acetyl CoA synthesis, as a representation of a high fat diet or comorbidity, and ageing on (**A**) [Acetyl CoA], (**B**) [FC], (**C**) [CE], (**D**) [LDLC], and (**E**) LDLr.

Due to the sensitivity of model species and reaction fluxes to perturbations in acetyl CoA synthesis, as outlined in the MCAs, changes to acetyl CoA synthesis were investigated. Values of 0.115, 0.175, and 0.2 were utilised for the rate constant K1 for acetyl CoA synthesis to represent a high fat diet, T2DM, and T1DM, respectively, as outlined by Perry et al. (2017). The baseline rate constant was assigned a value of 0.1. By editing this rate constant, acetyl CoA increased to 57.52 µmol/µL, approximately double the baseline acetyl CoA concentration of 32.13 µmol/µL (Figure 4A). Perturbed acetyl CoA synthesis was simulated in the presence or absence of aging. As the rate of acetyl CoA synthesis increases, there is a rise in FC, CE and LDL-C (Figure 4B–D). The rise in hepatic FC was greater in the aged simulation, whilst the rise in CE and LDL-C was less pronounced when aging was less pronounced in the aged simulation (Figure 4E).

## 4. Discussion

The dysregulation of cholesterol homeostasis, which results in elevated levels of LDL-C, is directly relevant to the pathogenesis of CVD [104]. Demographically it is well recognised that LDL-C levels rise with age until middle age in both males and females. Paradoxically, from a demographic perspective a decline in LDL-C from the midpoint of life onwards has also been observed [26]. Interestingly, when cholesterol metabolism has been examined in the oldest old, an intriguing inconsistency has been identified. Namely, in certain studies, low levels of LDL-C have been associated with an increased risk of mortality. In one study, which examined LDL-C levels in the oldest old, it was observed that a higher LDL-C level was associated with lower risk of all-cause mortality [105]. Moreover, other studies have shown that mortality from disease in old age is independent of both total cholesterol and LDL-C [30,106]. The reason for this remains unknown. To explore this puzzle, a computational model was used to investigate the intersection between hepatic cholesterol homeostasis and aging. Because ROS are inevitable byproducts of oxidative phosphorylation [107], they have been linked to the aging process [108]; therefore, it was meaningfully speculated that ROS have an impact on cholesterol metabolism. Our reason for this idea is underpinned by recent experimental evidence which has associated ROS with an increase in SREBP2 and HMGCR expression. This experimental work also demonstrated a concurrent rise in intracellular hepatic cholesterol and LDLr expression. Thus, ROS can not only affect the cholesterol biosynthesis pathway but also the hepatic cholesterol uptake [55]. The model was used to explore these experimental findings and our hypothesis by examining changes to HMGCR. The model responded to an increase in the V_max_ for HMGCR with a decline in LDLr. While this is biologically intuitive, this result differs from those found experimentally. This can be explained as HMGCR was investigated as a proxy for ROS, and ROS itself was not the investigated variable. In agreement with Seo et al. (2019), the model indicated that increased HMGCR activity resulted in elevated hepatic FC. Moreover, as the V_max_ of HMGCR increased, there was a marginal rise in the amount of cellular CE. These small changes are perhaps unsurprising because we and others have previously shown that there is an inherent robustness associated with cholesterol biosynthesis [83,84,85]. Although these increases were not overly significant and are in the main qualitative, they add some weight to the premise that if ROS augment HMGCR activity, there will be a potential increase FC. Perhaps more expectantly there was an increase in the concentration of LDL-C due to this perturbation.

The model was also used to investigate if an age-related decrease in hepatic ACAT2 activity can contribute to a reduction in plasma LDL-C levels. The rationale underpinning this idea is that hepatic ACAT2 catalyses the synthesis of CE available for secretion into nascent VLDL. Therefore, we hypothesised that this would lead to a concomitant decrease in plasma LDL-C, and a corresponding rise in hepatic FC. This is biologically possible because this phenomenon has been observed experimentally in older rabbits [58]. When this intervention was applied to the model intracellular FC increased, CE decreased and LDL-C decreased. Next the combined effects of both ROS and ACAT2 were investigated. This in silico experiment was conducted to investigate the cornerstone of our hypothesis; namely that these changes can account for the observation that low levels of LDL-C are associated with an increased risk of mortality in the oldest old [104]. It was found that the effects of ROS on the system were negligible, however, and significantly for our hypothesis it was found that as the activity of ACAT2 decreased, there was a concomitant decrease in plasma LDL-C. Thus, the model suggests a decrease in ACAT2 with age could contribute to a reduction in LDL-C, and presents a mechanistic explanation as to why low LDL-C have been observed in the oldest old [104]. This change also elicited an increase in FC. A build up in FC has cytotoxic implications because burgeoning clinical and experimental data suggest hepatic FC accumulation is directly relevant to the aetiology of NAFLD [109]. This finding is, to an extent, at odds with some recent experimental work which has suggested that the nonselective inhibition of ACAT2 could be a worthwhile way to treat hypercholesterolemia in humans [110]. This concept is largely fuelled by studies which have shown that ACAT2-deficient mice, when compared to wild type mice, have reduced hepatic CE and are protected against diet-induced atherosclerosis [111]. These experimental findings are intriguing and could be accounted for by inhibition of hepatic ACAT2 precipitating the decreased production of atherogenic apoB-containing lipoproteins, or inhibition of intestinal ACAT2 reducing cholesterol absorption [112].

The model also investigated the effect of increased flux of acetyl CoA. From a baseline value of 0.1, the rate constant for acetyl CoA synthesis was increased to 1.115, 1.175 and 0.2 to represent a high fat diet, poorly controlled T2DM and T1DM, respectively, as outlined by Perry et al. (2017). Model simulations revealed that elevated acetyl CoA synthesis were associated with an increase in LDL-C, FC, and CE and a decline in LDLr. Experimentally it has been shown that high fat diets are associated with hepatic fat accumulation. Accumulation of fat within the liver has been linked to an increase in PCSK9, an enzyme responsible for the degradation of LDLr. This resultant decline in cell-surface LDLr expression then impacts the rate of LDL-C clearance and LDL-C residence time, and is associated with a rise in LDL-C [113]. Interestingly, our model indicates that the changes outlined above are modulated by the aging process. The aged model was associated with greater hepatic FC and reduced plasma LDL-C accumulation. This can in part be explained by the hypothesis outlined.

## 5. Conclusions

To conclude, the model suggests that an increase in the activity of HMGCR does not have as significant an impact on cholesterol homeostasis as a decrease in hepatic ACAT2 activity. A decrease in the activity of hepatic ACAT2 raises hepatic free cholesterol (FC) and decreases LDL-C levels. The model also revealed that increased levels of acetyl CoA, which are linked with a high fat diet, are associated with increased LDL-C, FC and CE, in addition to reduced LDLr. Combining the effects of aging with increased acetyl CoA synthesis resulted in lower LDL-C which was associated with greater accumulation of hepatic FC. Taken together this has crucial implications for healthspan because emerging experimental and clinical data suggest hepatic FC accumulation is relevant to the pathogenesis of non-alcoholic fatty liver disease (NAFLD), which is associated with an increased risk of CVD. This pathophysiological change could in part help to explain the phenomenon of increased mortality associated with low levels of LDL-C, which have been observed in certain studies involving the oldest old (≥85 years).

## Figures and Tables

**Table 1:** List of Model Reactions *.

Reaction	Name	Abbreviation
R1	Acetyl coenzyme A (CoA) synthesis	CoAS → ACoA
R2	Interconversion of Acetyl CoA and Acetoacetyl CoA	ACoA = AACoA
R3	3-hydroxy-3-methylglutaryl (HMG)-CoA formation	ACoA + AACoA → HMGCoA
R4	Mevalonate (MV) formation	HMGCoA → MV
R5	Mevalonate5P (MV5P) formation	MV → MV5P
R6	Mevalonate5PP (MV5PP) formation	MV5P = MV5PP
R7	Isopentenyl-PP (IPP) formation	MV5PP → IPP
R8	Dimethylallyl-PP (DMAPP) interconversion	IPP = DMAPP
R9	GeranylPP (GPP) formation	DMAPP + IPP → GPP
R10	FarnesylPP (FPP) formation	GPP + IPP → FPP
R11	Squalene formation	FPP → SQ
R12	Squalene epoxide formation	SQ → SQE
R13	Lanosterol formation	SQE → LAN
R14	Free cholesterol (FC) formation	LAN → FC
R15	Conversion of FC to cholesteryl esters (CE)	FC → CE
R16	Conversion of CE to FC	CE → FC
R17	Cholesterol esters flux to low-density lipoprotein cholesterol (LDL-C)	CE → LDLC
R18	LDL-C sink	LDLC → LDLCs
R19	LDL receptor (LDLr) synthesis	sLDLR → LDLR
R20	LDLr degradation	LDLR → dLDLR
R21	Reuptake of LDL-C	LDLC → FC
R22	SREBP synthesis	sSRBP2 → SRBP2
R23	SREBP degradation	SRBP2 → dSRBP2
R24	Antioxidant production	sAOX → AOX
R25	Reactive oxygen species (ROS) production	sROS → ROS
R26	ROS degradation	AOX+ROS → ROSsink
R27	HMGCoA reductase synthesis	sHMGCoAR → HMGCoAR
R28	HMGCoA reductase degradation	HMGCoAR → dHMGCoAR
R29	Acetyl-CoA acetyltransferase 2 (ACAT2) synthesis	sACAT2 → ACAT2
R30	ACAT2 Degradation	ACAT2 → dACAT2

* See Table S1 in Supplementary File S2 for abbreviations.

**Table 2:** The Effect of combined HMGCR and ACAT2 modulation on FC, CE, LDL-C and LDLr.

V_max_ R15 Conversion of FC to CE (µMoles/min)	V_max_ R4 Mevalonate Formation (µMoles/min)				
	1	2	3	4	5
**FC**					
0.01	22.1333	22.2205	22.2485	22.2623	22.2705
0.02	9.93711	9.96287	9.97106	9.97509	9.97749
0.03	4.75843	4.76165	4.76266	4.76315	4.76345
0.04	2.83914	2.8396	2.83975	2.83981	2.83985
0.05	1.99568	1.99583	1.99587	1.99589	1.9959
**CE**					
0.01	0.084617	0.084669	0.084685	0.084693	0.084698
0.02	0.14247	0.142576	0.14261	0.142627	0.142637
0.03	0.162901	0.162953	0.162969	0.162976	0.162981
0.04	0.166003	0.166019	0.166024	0.166026	0.166028
0.05	0.166386	0.166394	0.166397	0.166398	0.166399
**LDL-C**					
0.01	0.08454	0.084591	0.084608	0.084616	0.084621
0.02	0.14232	0.142428	0.142462	0.142479	0.142489
0.03	0.162786	0.162839	0.162855	0.162863	0.162867
0.04	0.165925	0.165941	0.165947	0.165949	0.16595
0.05	0.166317	0.166326	0.166328	0.166329	0.16633
**LDLr**					
0.01	34,335.9	34,334	34,333.4	34,333.1	34,332.9
0.02	34,530.3	34,527	34,526	34,525.5	34,525.2
0.03	34,770.5	34,766.7	34,765.4	34,764.8	34,764.4
0.04	34,998.3	34,994.4	34,993.2	34,992.5	34,992.2
0.05	35,192	35,188.2	35,187	35,186.4	35,186

## Supplementary Materials

The following are available online at https://www.mdpi.com/2079-7737/9/10/314/s1, Supplementary File S1: SBGN diagram VANTED file; Supplementary File S2: Table S1, List of species and their full name, Table S2, Scaled concentration control coefficients, Table S3, Scaled flux control coefficients, Figure S1, Model variables reaching or approaching steady state, Figure S2, The Effect of combined HMGCR and ACAT2 modulation on A) [FC], B) [CE], C) [LDL-C] and D) LDLr; Supplementary File S3: a COPASI file of the model; Supplementary File S4: Model SBML; Supplementary File S5: An Excel file which provides the experimental sources of model parameters.

## References

[1] Homan R., Krause B.R. (1997). Established and emerging strategies for inhibition of cholesterol absorption. Curr. Pharm. Des..

[2] van der Wulp M.Y., Verkade H.J., Groen A.K. (2013). Regulation of cholesterol homeostasis. Mol. Cell. Endocrinol..

[3] Javitt N.B. (1994). Bile acid synthesis from cholesterol: Regulatory and auxiliary pathways. FASEB J..

[4] Payne A.H., Hales D.B. (2004). Overview of steroidogenic enzymes in the pathway from cholesterol to active steroid hormones. Endocr. Rev..

[5] Bikle D.D. (2014). Vitamin d metabolism, mechanism of action, and clinical applications. Chem. Biol..

[6] Castelli W.P., Anderson K., Wilson P.W., Levy D. (1992). Lipids and risk of coronary heart disease. The framingham study. Ann. Epidemiol..

[7] O’Donnell C.J., Elosua R. (2008). Cardiovascular risk factors. Insights from framingham heart study. Rev. Esp. Cardiol..

[8] Mc Auley M.T., Mooney K.M. (2014). Lipid metabolism and hormonal interactions: Impact on cardiovascular disease and healthy aging. Expert Rev. Endocrinol. Metab..

[9] Mooney K.M., Mc Auley M.T. (2016). Cardiovascular disease and healthy ageing. J. Integr. Cardiol..

[10] Fajemiroye J.O., Cunha L.C.d., Saavedra-Rodríguez R., Rodrigues K.L., Naves L.M., Mourão A.A., Silva E.F.d., Williams N.E.E., Martins J.L.R., Sousa R.B. (2018). Aging-induced biological changes and cardiovascular diseases. Biomed. Res. Int..

[11] Austin M.A., Breslow J.L., Hennekens C.H., Buring J.E., Willett W.C., Krauss R.M. (1988). Low-density lipoprotein subclass patterns and risk of myocardial infarction. JAMA.

[12] Ference B.A., Ginsberg H.N., Graham I., Ray K.K., Packard C.J., Bruckert E., Hegele R.A., Krauss R.M., Raal F.J., Schunkert H. (2017). Low-density lipoproteins cause atherosclerotic cardiovascular disease. 1. Evidence from genetic, epidemiologic, and clinical studies. A consensus statement from the european atherosclerosis society consensus panel. Eur. Heart J..

[13] Morgan A.E., Mooney K.M., Wilkinson S.J., Pickles N.A., Mc Auley M.T. (2016). Cholesterol metabolism: A review of how ageing disrupts the biological mechanisms responsible for its regulation. Ageing Res. Rev..

[14] Kreisberg R.A., Kasim S. (1987). Cholesterol metabolism and aging. Am. J. Med..

[15] Berrougui H., Khalil A. (2009). Age-associated decrease of high-density lipoprotein-mediated reverse cholesterol transport activity. Rejuvenation Res..

[16] Holzer M., Trieb M., Konya V., Wadsack C., Heinemann A., Marsche G. (2013). Aging affects high-density lipoprotein composition and function. Biochim. Biophys. Acta.

[17] Parini P., Angelin B., Rudling M. (1999). Cholesterol and lipoprotein metabolism in aging: Reversal of hypercholesterolemia by growth hormone treatment in old rats. Arterioscler. Thromb. Vasc. Biol..

[18] Uchida K., Nomura Y., Kadowaki M., Takase H., Takano K., Takeuchi N. (1978). Age-related changes in cholesterol and bile acid metabolism in rats. J. Lipid Res..

[19] Liu H.H., Li J.J. (2015). Aging and dyslipidemia: A review of potential mechanisms. Ageing Res. Rev..

[20] Bertolotti M., Abate N., Bertolotti S., Loria P., Concari M., Messora R., Carubbi F., Pinetti A., Carulli N. (1993). Effect of aging on cholesterol 7 alpha-hydroxylation in humans. J. Lipid Res..

[21] Mc Auley M., Jones J., Wilkinson D., Kirkwood T. (2005). Modelling lipid metabolism to improve healthy ageing. BMC Bioinform..

[22] Carroll M.D., Lacher D.A., Sorlie P.D., Cleeman J.I., Gordon D.J., Wolz M., Grundy S.M., Johnson C.L. (2005). Trends in serum lipids and lipoproteins of adults, 1960–2002. JAMA.

[23] Wang M., Hou X., Hu W., Chen L., Chen S. (2019). Serum lipid and lipoprotein levels of middle-aged and elderly chinese men and women in shandong province. Lipids Health Dis..

[24] Farzadfar F., Finucane M.M., Danaei G., Pelizzari P.M., Cowan M.J., Paciorek C.J., Singh G.M., Lin J.K., Stevens G.A., Riley L.M. (2011). National, regional, and global trends in serum total cholesterol since 1980: Systematic analysis of health examination surveys and epidemiological studies with 321 country-years and 3.0 million participants. Lancet.

[25] Duncan M.S., Vasan R.S., Xanthakis V. (2019). Trajectories of blood lipid concentrations over the adult life course and risk of cardiovascular disease and all-cause mortality: Observations from the framingham study over 35 years. J. Am. Heart Assoc..

[26] Felix-Redondo F.J., Grau M., Fernandez-Berges D. (2013). Cholesterol and cardiovascular disease in the elderly. Facts and gaps. Aging Dis..

[27] Ferrara A., Barrett-Connor E., Shan J. (1997). Total, ldl, and hdl cholesterol decrease with age in older men and women. The rancho bernardo study 1984–1994. Circulation.

[28] Garry P.J., Hunt W.C., Koehler K.M., VanderJagt D.J., Vellas B.J. (1992). Longitudinal study of dietary intakes and plasma lipids in healthy elderly men and women. Am. J. Clin. Nutr..

[29] Postmus I., Deelen J., Sedaghat S., Trompet S., de Craen A.J., Heijmans B.T., Franco O.H., Hofman A., Dehghan A., Slagboom P.E. (2015). Ldl cholesterol still a problem in old age? A mendelian randomization study. Int. J. Epidemiol..

[30] Weverling-Rijnsburger A.W., Jonkers I.J., van Exel E., Gussekloo J., Westendorp R.G. (2003). High-density vs. low-density lipoprotein cholesterol as the risk factor for coronary artery disease and stroke in old age. Arch. Intern. Med..

[31] Ravnskov U. (2003). High cholesterol may protect against infections and atherosclerosis. QJM.

[32] Han R. (2010). Plasma lipoproteins are important components of the immune system. Microbiol. Immunol..

[33] Ravnskov U., de Lorgeril M., Diamond D.M., Hama R., Hamazaki T., Hammarskjold B., Hynes N., Kendrick M., Langsjoen P.H., Mascitelli L. (2018). Ldl-c does not cause cardiovascular disease: A comprehensive review of the current literature. Expert Rev. Clin. Pharmacol..

[34] Ravnskov U., Diamond D.M., Hama R., Hamazaki T., Hammarskjöld B., Hynes N., Kendrick M., Langsjoen P.H., Malhotra A., Mascitelli L. (2016). Lack of an association or an inverse association between low-density-lipoprotein cholesterol and mortality in the elderly: A systematic review. BMJ Open.

[35] Mc Auley M.T., Mooney K.M. (2017). Ldl-c levels in older people: Cholesterol homeostasis and the free radical theory of ageing converge. Med. Hypotheses.

[36] Mc Auley M.T. (2018). The interplay between cholesterol metabolism and intrinsic ageing. Subcell. Biochem..

[37] Tiwari S., Siddiqi S.A. (2012). Intracellular trafficking and secretion of vldl. Arterioscler. Thromb. Vasc. Biol..

[38] Goldberg I.J. (1996). Lipoprotein lipase and lipolysis: Central roles in lipoprotein metabolism and atherogenesis. J. Lipid Res..

[39] Goldstein J.L., Brown M.S. (2009). The ldl receptor. Arterioscler. Thromb. Vasc. Biol..

[40] Brown M.S., Radhakrishnan A., Goldstein J.L. (2018). Retrospective on cholesterol homeostasis: The central role of scap. Annu. Rev. Biochem..

[41] Sato R. (2010). Sterol metabolism and srebp activation. Arch. Biochem. Biophys..

[42] Eberlé D., Hegarty B., Bossard P., Ferré P., Foufelle F. (2004). Srebp transcription factors: Master regulators of lipid homeostasis. Biochimie.

[43] Jeong H.J., Lee H.-S., Kim K.-S., Kim Y.-K., Yoon D., Park S.W. (2008). Sterol-dependent regulation of proprotein convertase subtilisin/kexin type 9 expression by sterol-regulatory element binding protein-2. J. Lipid Res..

[44] Lagace T.A. (2014). Pcsk9 and ldlr degradation: Regulatory mechanisms in circulation and in cells. Curr. Opin. Lipidol..

[45] Reiss A.B., Shah N., Muhieddine D., Zhen J., Yudkevich J., Kasselman L.J., DeLeon J. (2018). Pcsk9 in cholesterol metabolism: From bench to bedside. Clin. Sci..

[46] Chaudhary R., Garg J., Shah N., Sumner A. (2017). Pcsk9 inhibitors: A new era of lipid lowering therapy. World J. Cardiol..

[47] Parini P., Davis M., Lada A.T., Erickson S.K., Wright T.L., Gustafsson U., Sahlin S., Einarsson C., Eriksson M., Angelin B. (2004). Acat2 is localized to hepatocytes and is the major cholesterol-esterifying enzyme in human liver. Circulation.

[48] Semsei I., Rao G., Richardson A. (1989). Changes in the expression of superoxide dismutase and catalase as a function of age and dietary restriction. Biochem. Biophys. Res. Commun..

[49] Ji L.L. (1993). Antioxidant enzyme response to exercise and aging. Med. Sci. Sports Exerc..

[50] Pallottini V., Martini C., Bassi A.M., Romano P., Nanni G., Trentalance A. (2006). Rat hmgcoa reductase activation in thioacetamide-induced liver injury is related to an increased reactive oxygen species content. J. Hepatol..

[51] Pallottini V., Martini C., Cavallini G., Bergamini E., Mustard K.J., Hardie D.G., Trentalance A. (2007). Age-related hmg-coa reductase deregulation depends on ros-induced p38 activation. Mech. Ageing Dev..

[52] Pallottini V., Martini C., Pascolini A., Cavallini G., Gori Z., Bergamini E., Incerpi S., Trentalance A. (2005). 3-hydroxy-3-methylglutaryl coenzyme a reductase deregulation and age-related hypercholesterolemia: A new role for ros. Mech. Ageing Dev..

[53] Trapani L., Pallottini V. (2010). Age-related hypercholesterolemia and hmg-coa reductase dysregulation: Sex does matter (a gender perspective). Curr. Gerontol. Geriatr. Res..

[54] Trapani L., Violo F., Pallottini V. (2010). Hypercholesterolemia and 3-hydroxy-3-methylglutaryl coenzyme a reductase regulation in aged female rats. Exp. Gerontol..

[55] Seo E., Kang H., Choi H., Choi W., Jun H.S. (2019). Reactive oxygen species-induced changes in glucose and lipid metabolism contribute to the accumulation of cholesterol in the liver during aging. Aging Cell.

[56] Mulas M.F., Demuro G., Mulas C., Putzolu M., Cavallini G., Donati A., Bergamini E., Dessi S. (2005). Dietary restriction counteracts age-related changes in cholesterol metabolism in the rat. Mech. Ageing Dev..

[57] Ståhlberg D., Angelin B., Einarsson K. (1991). Age-related changes in the metabolism of cholesterol in rat liver microsomes. Lipids.

[58] Shiomi M., Ito T., Fujioka T., Tsujita Y. (2000). Age-associated decrease in plasma cholesterol and changes in cholesterol metabolism in homozygous watanabe heritable hyperlipidemic rabbits. Metabolism.

[59] Ioannou G.N. (2016). The role of cholesterol in the pathogenesis of nash. Trends Endocrinol. Metab..

[60] Hagstrom H., Nasr P., Ekstedt M., Hammar U., Stal P., Askling J., Hultcrantz R., Kechagias S. (2019). Cardiovascular risk factors in non-alcoholic fatty liver disease. Liver Int..

[61] Morgan A.E., Mooney K.M., Wilkinson S.J., Pickles N.A., Mc Auley M.T. (2017). Investigating cholesterol metabolism and ageing using a systems biology approach. Proc. Nutr. Soc..

[62] Mc Auley M.T., Mooney K.M. (2015). Computationally modeling lipid metabolism and aging: A mini-review. Comput. Struct. Biotechnol. J..

[63] Mc Auley M., Mooney K., Ram J.L., Conn P.M. (2018). Chapter 7—Using computational models to study aging. Conn’s Handbook of Models for Human Aging.

[64] Neumann S.J., Berceli S.A., Sevick E.M., Lincoff A.M., Warty V.S., Brant A.M., Herman I.M., Borovetz H.S. (1990). Experimental determination and mathematical model of the transient incorporation of cholesterol in the arterial wall. Bull. Math Biol..

[65] Lu J., Hubner K., Nanjee M.N., Brinton E.A., Mazer N.A. (2014). An in-silico model of lipoprotein metabolism and kinetics for the evaluation of targets and biomarkers in the reverse cholesterol transport pathway. PLoS Comput. Biol..

[66] Mc Auley M.T., Kenny R.A., Kirkwood T.B., Wilkinson D.J., Jones J.J., Miller V.M. (2009). A mathematical model of aging-related and cortisol induced hippocampal dysfunction. BMC Neurosci..

[67] Fabregat A., Sidiropoulos K., Garapati P., Gillespie M., Hausmann K., Haw R., Jassal B., Jupe S., Korninger F., McKay S. (2016). The reactome pathway knowledgebase. Nucleic Acids Res..

[68] Kanehisa M., Furumichi M., Tanabe M., Sato Y., Morishima K. (2017). Kegg: New perspectives on genomes, pathways, diseases and drugs. Nucleic Acids Res..

[69] Mc Auley M.T., Guimera A.M., Hodgson D., McDonald N., Mooney K.M., Morgan A.E., Proctor C.J. (2017). Modelling the molecular mechanisms of aging. Biosci. Rep..

[70] Mooney K.M., Morgan A.E., Mc Auley M.T. (2016). Aging and computational systems biology. Wiley interdisciplinary reviews. Syst. Biol. Med..

[71] Mc Auley M.T., Proctor C.J., Corfe B.M., Cuskelly G.J., Mooney K.M. (2013). Nutrition research and the impact of computational systems biology. J. Comput. Sci. Syst. Biol..

[72] Mc Auley M.T., Mooney K.M., Angell P.J., Wilkinson S.J. (2015). Mathematical modelling of metabolic regulation in aging. Metabolites.

[73] Kilner J., Corfe B.M., McAuley M.T., Wilkinson S.J. (2016). A deterministic oscillatory model of microtubule growth and shrinkage for differential actions of short chain fatty acids. Mol. Biosyst..

[74] Mc Auley M.T., Choi H., Mooney K., Paul E., Miller V.M. (2015). Systems biology and synthetic biology: A new epoch for toxicology research. Adv. Toxicol..

[75] Saqi M., Pellet J., Roznovat I., Mazein A., Ballereau S., De Meulder B., Auffray C. (2016). Systems medicine: The future of medical genomics, healthcare, and wellness. Methods Mol. Biol. (Clifton N.J.).

[76] Ostaszewski M., Gebel S., Kuperstein I., Mazein A., Zinovyev A., Dogrusoz U., Hasenauer J., Fleming R.M.T., Le Novère N., Gawron P. (2019). Community-driven roadmap for integrated disease maps. Brief. Bioinform..

[77] Mc Auley M.T., Mooney K.M. (2015). Computational systems biology for aging research. Interdiscip. Top. Gerontol..

[78] Parton A., McGilligan V., O’Kane M., Baldrick F.R., Watterson S. (2016). Computational modelling of atherosclerosis. Brief. Bioinform..

[79] Mc Auley M.T., Wilkinson D.J., Jones J.J., Kirkwood T.B. (2012). A whole-body mathematical model of cholesterol metabolism and its age-associated dysregulation. BMC Syst. Biol..

[80] Pool F., Currie R., Sweby P.K., Salazar J.D., Tindall M.J. (2018). A mathematical model of the mevalonate cholesterol biosynthesis pathway. J. Theor. Biol..

[81] Bhattacharya B.S., Sweby P.K., Minihane A.M., Jackson K.G., Tindall M.J. (2014). A mathematical model of the sterol regulatory element binding protein 2 cholesterol biosynthesis pathway. J. Theor. Biol..

[82] Watterson S., Guerriero M.L., Blanc M., Mazein A., Loewe L., Robertson K.A., Gibbs H., Shui G., Wenk M.R., Hillston J. (2013). A model of flux regulation in the cholesterol biosynthesis pathway: Immune mediated graduated flux reduction versus statin-like led stepped flux reduction. Biochimie.

[83] Morgan A., Mooney K.M., Wilkinson S.J., Pickles N., Mc Auley M.T. (2016). Mathematically modelling the dynamics of cholesterol metabolism and ageing. Biosystems.

[84] Tindall M.J., Wattis J.A., O’Malley B.J., Pickersgill L., Jackson K.G. (2009). A continuum receptor model of hepatic lipoprotein metabolism. J. Theor. Biol..

[85] August E., Parker K.H., Barahona M. (2007). A dynamical model of lipoprotein metabolism. Bull. Math Biol..

[86] Pool F., Sweby P., Tindall M.J.P. (2018). An integrated mathematical model of cellular cholesterol biosynthesis and lipoprotein metabolism. Processes.

[87] Toroghi M.K., Cluett W.R., Mahadevan R.J.C., Engineering C. (2019). A multi-scale model for low-density lipoprotein cholesterol (ldl-c) regulation in the human body: Application to quantitative systems pharmacology. Comput. Chem. Eng..

[88] Kervizic G., Corcos L. (2008). Dynamical modeling of the cholesterol regulatory pathway with boolean networks. BMC Syst. Biol..

[89] Benson H.E., Watterson S., Sharman J.L., Mpamhanga C.P., Parton A., Southan C., Harmar A.J., Ghazal P. (2017). Is systems pharmacology ready to impact upon therapy development? A study on the cholesterol biosynthesis pathway. Br. J. Pharmacol..

[90] Mazein A., Watterson S., Hsieh W.Y., Griffiths W.J., Ghazal P. (2013). A comprehensive machine-readable view of the mammalian cholesterol biosynthesis pathway. Biochem. Pharmacol..

[91] Bourgin M., Labarthe S., Kriaa A., Lhomme M., Gérard P., Lesnik P., Laroche B., Maguin E., Rhimi M. (2020). Exploring the bacterial impact on cholesterol cycle: A numerical study. Front. Microbiol..

[92] Gomez-Cabrero D., Compte A., Tegner J. (2011). Workflow for generating competing hypothesis from models with parameter uncertainty. Interface Focus..

[93] Parton A., McGilligan V., Chemaly M., O’Kane M., Watterson S. (2019). New models of atherosclerosis and multi-drug therapeutic interventions. Bioinformatics.

[94] Bekkar A., Estreicher A., Niknejad A., Casals-Casas C., Bridge A., Xenarios I., Dorier J., Crespo I. (2018). Expert curation for building network-based dynamical models: A case study on atherosclerotic plaque formation. Database.

[95] Le Novere N., Hucka M., Mi H., Moodie S., Schreiber F., Sorokin A., Demir E., Wegner K., Aladjem M.I., Wimalaratne S.M. (2009). The systems biology graphical notation. Nat. Biotechnol..

[96] Junker B.H., Klukas C., Schreiber F. (2006). Vanted: A system for advanced data analysis and visualization in the context of biological networks. BMC Bioinform..

[97] Czauderna T., Klukas C., Schreiber F. (2010). Editing, validating and translating of sbgn maps. Bioinformatics.

[98] Wildermuth M.C. (2000). Metabolic control analysis: Biological applications and insights. Genome Biol..

[99] Mc Auley M.T. (2019). Model analysis in greater depth. Computer Modelling for Nutritionists.

[100] Fell D., Cornish-Bowden A. (1997). Understanding the Control of Metabolism.

[101] Shi L., Tu B.P. (2015). Acetyl-coa and the regulation of metabolism: Mechanisms and consequences. Curr. Opin. Cell Biol..

[102] Perry R.J., Peng L., Cline G.W., Petersen K.F., Shulman G.I. (2017). A non-invasive method to assess hepatic acetyl-coa in vivo. Cell Metab..

[103] Johnston T.P., Palmer W.K. (1997). The effect of pravastatin on hepatic 3-hydroxy-3-methylglutaryl coa reductase obtained from poloxamer 407-induced hyperlipidemic rats. Pharmacotherapy.

[104] Pedersen T.R. (2016). The success story of ldl cholesterol lowering. Circ. Res..

[105] Lv Y.B., Yin Z.X., Chei C.L., Qian H.Z., Kraus V.B., Zhang J., Brasher M.S., Shi X.M., Matchar D.B., Zeng Y. (2015). Low-density lipoprotein cholesterol was inversely associated with 3-year all-cause mortality among chinese oldest old: Data from the chinese longitudinal healthy longevity survey. Atherosclerosis.

[106] Weverling-Rijnsburger A.W., Blauw G.J., Lagaay A.M., Knook D.L., Meinders A.E., Westendorp R.G. (1997). Total cholesterol and risk of mortality in the oldest old. Lancet.

[107] Al-Mallah M.H., Hatahet H., Cavalcante J.L., Khanal S. (2009). Low admission ldl-cholesterol is associated with increased 3-year all-cause mortality in patients with non st segment elevation myocardial infarction. Cardiol. J..

[108] Liguori I., Russo G., Curcio F., Bulli G., Aran L., Della-Morte D., Gargiulo G., Testa G., Cacciatore F., Bonaduce D. (2018). Oxidative stress, aging, and diseases. Clin. Interv. Aging.

[109] Tirosh O. (2018). Hypoxic signaling and cholesterol lipotoxicity in fatty liver disease progression. Oxid. Med. Cell. Longev..

[110] Chang T.Y., Li B.L., Chang C.C., Urano Y. (2009). Acyl-coenzyme a: Cholesterol acyltransferases. Am. J. Physiol. Endocrinol. Metab..

[111] Bell T.A., Brown J.M., Graham M.J., Lemonidis K.M., Crooke R.M., Rudel L.L. (2006). Liver-specific inhibition of acyl-coenzyme a: Cholesterol acyltransferase 2 with antisense oligonucleotides limits atherosclerosis development in apolipoprotein b100-only low-density lipoprotein receptor-/- mice. Arterioscler. Thromb. Vasc. Biol..

[112] Temel R.E., Lee R.G., Kelley K.L., Davis M.A., Shah R., Sawyer J.K., Wilson M.D., Rudel L.L. (2005). Intestinal cholesterol absorption is substantially reduced in mice deficient in both abca1 and acat2. J. Lipid Res..

[113] Lebeau P.F., Byun J.H., Platko K., MacDonald M.E., Poon S.V., Faiyaz M., Seidah N.G., Austin R.C. (2019). Diet-induced hepatic steatosis abrogates cell-surface ldlr by inducing de novo pcsk9 expression in mice. J. Biol. Chem..

