# Supplementary material for: Cholesterol Homeostasis: An In Silico Investigation into How Aging Disrupts Its Key Hepatic Regulatory Mechanisms
Source: Biology (Basel). 2020 Sep 30;9(10):314. doi: 10.3390/biology9100314 (PMC7599957; doi:10.3390/biology9100314)
Supplement: Supplementary file 1 [file biology-09-00314-s001.zip › biology-931909-supplementary/Supplementary Files/Supplementary File 2.docx]

**Table 1. List of species and their full name.**

| **Species** | **Full Name** |
| --- | --- |
| CoAS | Acetyl CoA synthesis |
| ACoA | Acetyl CoA |
| AACoA | AcetoAcetyl CoA |
| HMGCoA | HMG CoA |
| MV5P | Mevalonate5P |
| MV5PP | Mevalonate5PP |
| IPP | Isopentenyl pyrophosphate |
| DMAPP | Dimethylallyl pyrophosphate |
| GPP | GeranylPP |
| FPP | FarnesylPP |
| SQ | Squalene |
| SQE | Squalene epoxide |
| LAN | Lanosterol |
| FC | Free Cholesterol |
| CE | Cholesteryl esters |
| LDLC | Low Density Lipoprotein-Cholesterol |
| LDLCs | LDL-C synthesis |
| LDLR | Low density lipoprotein Receptor |
| sLDLR | LDLR synthesis |
| dLDLR | LDLR degradation |
| SRBP2 | Sterol regulatory element-binding protein 2 |
| sSRBP2 | Sterol regulatory element-binding protein 2 synthesis |
| dSRBP2 | SREBP degradation |
| sAOX | Antioxidant production |
| AOX | Antioxidants |
| ROS | Reactive oxygen species |
| sROS | ROS Production |
| ROSsink | ROS Degradation |
| sHMGCoAR | HMCoA Reducaste Synthesis |
| HMGCoAR | HMCoA Reductase |
| dHMGCoAR | HMCoA Reductase degradation |
| sACAT | Acyl-CoA cholesterol acyltransferase 2 synthesis |
| ACAT2 | Acyl-CoA cholesterol acyltransferase 2 |
| dACAT2 | Acyl-CoA cholesterol acyltransferase 2 degradation |

**
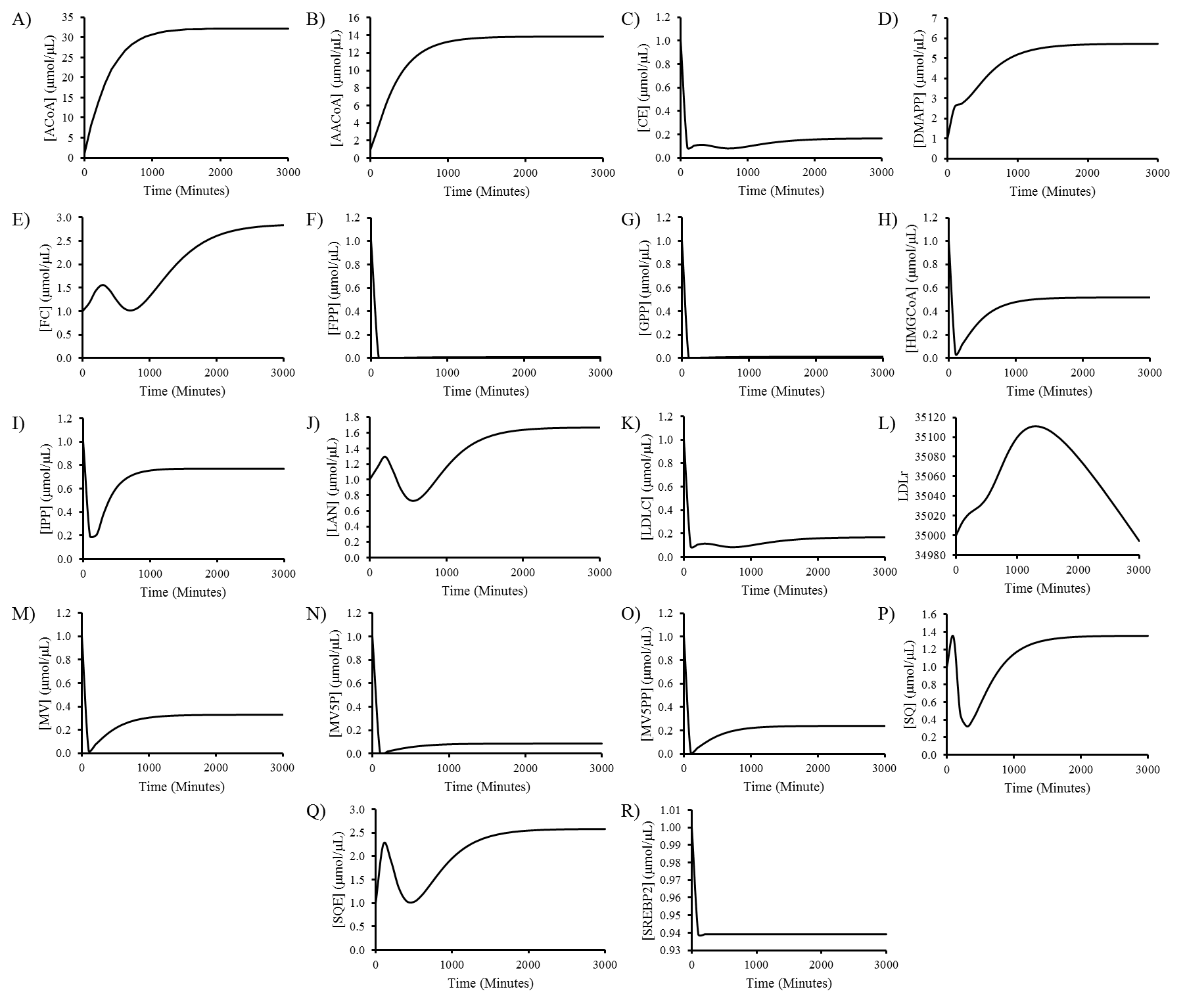
**

**Figure 1. Model variables reaching or approaching steady state**: A) [ACoA], B) [AACoA], C) [CE], D) [DMAPP], E) [FC], F) [FPP], G) [GPP], H) [HMGCoA], I) [IPP], J) [LAN], K) [LDLC], L) LDLr, M) [MV], N) [MV5P], O) [MV5PP], P) [SQ], Q) [SQE], and R) [SREBP2].

**Table S3 Scaled flux control coefficients.**

**Table S2 Scaled concentration control coefficients.**


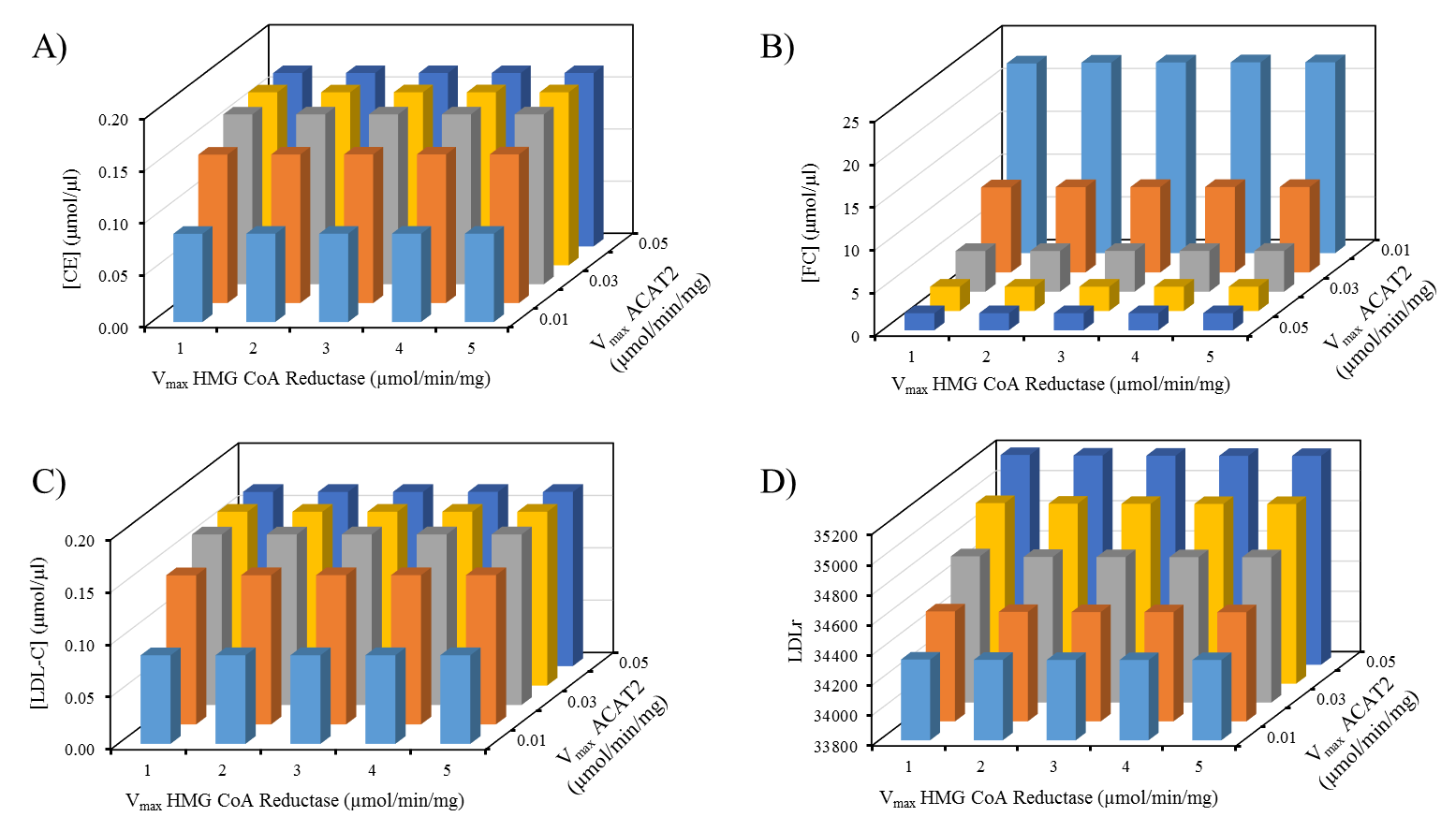


**Figure S2.** **The Effect of combined HMGCR and ACAT2 modulation on A) [FC], B) [CE], C) [LDL-C] and D) LDLr**.There is a negligible increase in [FC] as V_max_ for HMGCR is increased, while an increase in ACAT2 has a more pronounced effect on [FC].There is a negligible increase in [CE] as V_max_ for HMGCR is increased, while an increase in ACAT2 significantly reduces [CE].There is a negligible increase in [LDL-C] as V_max_ for HMGCR is increased, while an increase in ACAT2 has a more pronounced effect on [LDL-C]. There is a negligible decrease in LDLr as V_max_ for HMGCR is increased, while an increase in ACAT2 has a more pronounced effect on LDLr.
